# Can we *S*ave the rectum by watchful waiting or *T*rans*A*nal microsurgery following (chemo) *R*adiotherapy versus *T*otal mesorectal excision for early *RE*ctal *C*ancer (STAR-TREC study)?: protocol for a multicentre, randomised feasibility study

**DOI:** 10.1136/bmjopen-2017-019474

**Published:** 2017-12-28

**Authors:** Anouk J M Rombouts, Issam Al-Najami, Natalie L Abbott, Ane Appelt, Gunnar Baatrup, Simon Bach, Aneel Bhangu, Karen-Lise Garm Spindler, Richard Gray, Kelly Handley, Manjinder Kaur, Ellen Kerkhof, Camilla Jensenius Kronborg, Laura Magill, Corrie A M Marijnen, Iris D Nagtegaal, Lars Nyvang, Femke P Peters, Per Pfeiffer, Cornelis Punt, Philip Quirke, David Sebag-Montefiore, Mark Teo, Nick West, Johannes H W de Wilt

**Affiliations:** 1 Department of Surgery, Radboud University Medical Centre, Nijmegen, The Netherlands; 2 Department of Surgery, Odense University Hospital, Odense, Denmark; 3 Radiotheraphy Trials Quality Assurance Group, Velindre Cancer Centre, Cardiff, UK; 4 Leeds Institute of Cancer and Pathology, University of Leeds, Leeds, UK; 5 Leeds Cancer Centre, St. James’ University Hospital, Leeds, UK; 6 Department of Surgery, University Hospitals Birmingham, Birmingham, UK; 7 Department of Clinical Oncology, Aarhus University Hospital, Aarhus, Denmark; 8 Department of Clinical Medicine, Aarhus University, Aarhus, Denmark; 9 Clinical Trial Services Unit, University of Oxford, Oxford, UK; 10 Institue of Applied Health Research, University of Birmingham Clinical Trials Unit, Birmingham, UK; 11 Birmingham Clinical Trials Unit, University of Birmingham, Birmingham, UK; 12 Department of Radiotherapy, Leiden University Medical Center, Leiden, The Netherlands; 13 Department of Clinical Oncology, Aarhus University Hospital, Aarhus, Denmark; 14 Department of Pathology, Radboud University Medical Centre, Nijmegen, The Netherlands; 15 Department of Medical Physics, Aarhus University Hospital, Aarhus, Denmark; 16 Department of Oncology, Odense University Hospital, Odense, Denmark; 17 Department of Medical Oncology, Academic Medical Center, Amsterdam, The Netherlands; 18 Department of Pathology, School of Medicine, University of Leeds, Leeds, UK; 19 Department of Clinical Oncology, Leeds Radiotherapy Research Group, University of Leeds, Leeds, UK; 20 Department of Clinical Oncology, Leeds Cancer Centre, St James University Hospital, Leeds, UK

**Keywords:** rectal cancer, chemoradiation, TEM, radiotherapy, watchful waiting

## Abstract

**Introduction:**

Total mesorectal excision (TME) is the highly effective standard treatment for rectal cancer but is associated with significant morbidity and may be overtreatment for low-risk cancers. This study is designed to determine the feasibility of international recruitment in a study comparing organ-saving approaches versus standard TME surgery.

**Methods and analysis:**

STAR-TREC trial is a multicentre international randomised, three-arm parallel, phase II feasibility study in patients with biopsy-proven adenocarcinoma of the rectum. The trial is coordinated from Birmingham, UK with national hubs in Radboudumc (the Netherlands) and Odense University Hospital Svendborg UMC (Denmark). Patients with rectal cancer, staged by CT and MRI as ≤cT3b (up to 5 mm of extramural spread) N0 M0 can be included. Patients will be randomised to either standard TME surgery (control), organ-saving treatment using long-course concurrent chemoradiation or organ-saving treatment using short-course radiotherapy. For patients treated with an organ-saving strategy, clinical response to (chemo)radiotherapy determines the next treatment step. An active surveillance regime will be performed in the case of a complete clinical regression. In the case of incomplete clinical regression, patients will proceed to local excision using an optimised platform such as transanal endoscopic microsurgery or other transanal techniques (eg, transanal endoscopic operation or transanal minimally invasive surgery). The primary endpoint of this phase II study is to demonstrate sufficient international recruitment in order to sustain a phase III study incorporating pelvic failure as the primary endpoint. Success in phase II is defined as randomisation of at least four cases per month internationally in year 1, rising to at least six cases per month internationally during year 2.

**Ethics and dissemination:**

The medical ethical committees of all the participating countries have approved the study protocol. Results of the primary and secondary endpoints will be submitted for publication in peer-reviewed journals.

**Trial registration number:**

ISRCTN14240288, 20 October 2016. NCT02945566; Pre-results, October 2016.

**Strengths and limitations of this study**

- This phase II study is the first study to randomise between the standard of care in early rectal cancer (ie, total mesorectal excision surgery) and two organ-saving strategies using (chemo)radiotherapy followed by selective transanal microsurgery.
- STAR-TREC study will show whether it is feasible to recruit enough patients for a consecutive international large, multicentre randomised phase III trial.
- The study design incorporates several adjustments in standard (chemo)radiation therapy protocols intended to reduce treatment-related side effects associated with organ-saving therapy.
- Clinical nodal staging of rectal cancer is rather unreliable and patients with false-negative nodal disease will be included in the study.
- Experience with clinical judgement of a complete response is difficult and needs to be monitored carefully with central reviewing during the study.

## Introduction

The introduction of bowel cancer screening is associated with a significant increase in the incidence of early-stage rectal cancer.^1 2^ Total mesorectal excision (TME) surgery is an effective oncological treatment for early-stage rectal cancer, only 2% and 12% of patients experience local or distant failure, respectively.^3–5^ However, standard surgery for rectal cancer requires permanent stoma formation in 10%–20% of cases and temporary stoma formation in 60%–70%.^6 7^ Many temporary stomas are not reversed.^8 9^ Furthermore, TME surgery is associated with major morbidity and mortality in a significant number of patients. Over 50% of all patients following TME surgery experience faecal incontinence, whereas urinary problems and sexual dysfunction are observed in 32%–80% of patients.^10–14^ Another complication following TME surgery is anastomotic bowel leakage which occurs in approximately 15% of patients.^15^ In addition, quality-of-life studies show that TME surgery is associated with persistently poor social role and body image.^12 16–19^ Mortality following TME surgery rises with age; the 6-month mortality following TME surgery is 2.0%–4.6% for young patients with rectal cancer and 9.0%–13.4% for elderly patients (aged >75 years).^20–22^ There are concerns that TME surgery, which evolved to treat locally advanced, symptomatic tumours, may result in significant overtreatment of early screen-detected tumours. An organ-preserving strategy may generate significantly less morbidity without substantially compromising oncological outcomes. Promising outcomes have been reported for (chemo)radiation therapy followed by watchful waiting or local excision.

Habr-Gama’s group have notably published a watchful waiting approach to rectal cancer. Of 265 patients with predominantly T3 rectal cancer treated with chemoradiation therapy (CRT), 71 patients (27%) had a complete clinical response (cCR).^23 24^ These patients did not have surgery and after a mean follow-up of 57 months (range: 18–156), only two patients developed local recurrence, one of which was successfully salvaged. A Dutch group then prospectively selected patients with cCR for a watchful waiting strategy (n=21).^25^ After a mean follow-up 25 months (±19 months), one patient had developed a local recurrence which was salvaged by surgery and all other patients were alive without disease. In 2015, the effect of a radiation boost after CRT was evaluated in a prospective observational Danish study. A watch and wait policy was possible in 40 out of 51 included patients.^26^ At 1 year, local recurrence occurred in 16% of 40 patients who initially showed a cCR. Rectal bleeding was relatively frequent in this study during follow-up perhaps relating to the higher radiotherapy doses that were used. However, these results which combine high cCR rates and low local recurrence rates have not been consistently replicated.^27 28^ Furthermore, CRT is associated with treatment-related morbidity and a mortality rate of 0.5%–1% should be considered.^29^

Another organ-saving treatment strategy is local excision instead of radical surgery. Early rectal tumours may be locally excised through the anus with low morbidity and mortality using transanal endoscopic microsurgery (TEM), allowing rectal-saving treatment.^30 31^ Morbidity and mortality after local excision are much lower than after major resection. Morbidity associated with TEM includes bowel perforation, (transitory) incontinence, wound infection and local pain.^32–34^ In a study of 5305 patients with early-stage rectal cancer, 30-day mortality after local excision was found to be 0.5% compared with 2.4% in patients undergoing major resection (P=0.008). Morbidity within 30 days of surgery was 4.4% in the local excision group versus 12.7% in the major resection group (P<0.001).^34^ However, the risk of non-radical resection after local excision is higher and the risks of leaving behind microscopic lymph node metastases are a potential cause of local failure.^5 35^ The incidence of lymph node metastasis ranges from 6% to 14% for T1 tumours, 17% to 23% for T2 tumours and 49% to 66% for T3 tumours.^36^

Combining radiotherapy with TEM could possibly lead to better outcomes because radiotherapy can effectively treat microscopic mesorectal nodal metastases and contribute to tumour downsizing.^37 38^ However, limited prospective evidence currently exists to guide the use of radiotherapy and local excision as curative treatment for early rectal cancer. Lezoche *et al* randomised 100 patients with T2N0 rectal cancer to CRTfollowed by laparoscopic TME surgery or CRT and TEM with a 6–8 week interval to surgery.^39^ After a median follow-up of 9 years, local recurrence rates were 6% and 8% in the TME and TEM arms, respectively. In a trial of 89 patients with unfavourable cT1N0, cT2N0 or borderline cT2/3N0 tumours by Bujko *et al*, patients were given neoadjuvant treatment with short-course radiation therapy (SCRT) or CRT prior to delayed local excision.^40^ No further treatment was offered for good responders, whereas immediate TME surgery was recommended for all other patients. Good responders had a 2-year local recurrence rate of 10%. Of the poor responders, eight patients had a TME and none of this group had a recurrence, however, 18 declined or were unfit for TME, and this group had a 2-year local recurrence rate of 37%. This underlines that in high-risk or poor responding patients, neoadjuvant radiotherapy followed by local excision is inadequate treatment.

A study investigating chemoradiation therapy for rectal cancer in the distal rectum followed by organ-sparing transanal endoscopic microsurgery (CARTS) was a non-randomised phase II study that evaluated CRT followed by TEM in 55 patients with stage T1–3N0 rectal cancer.^41^ Clinical response was assessed 6–8 weeks after completion of CRT and TEM was performed. Organ saving was achieved in more than half of patients and 21 had ypT0 disease. Radiotherapy consisted of 50 Gy in 25 fractions and capecitabine 825 mg/m^2^ two times per day was given for the same period 7 days per week. However, 42% of patients developed at least grade 3 toxicity and there were two toxicity-related deaths. A multicentre cohort study from the UK that employed SCRT with TEM after 10 weeks demonstrated that 43/62 cases had either no or minimal residual disease following radiotherapy.^42^ None of these patients experienced short-term pelvic relapse and treatment-related toxicity was low. The ACOSOG Z6041 study, a single-arm phase II study, evaluated an oxaliplatin and capecitabine concurrent chemotherapy schedule combined with 54 Gy of pelvic radiotherapy followed by TEM for T2N0 rectal cancer.^43^ Both radiotherapy and chemotherapy schedules required reduction during the study due to acute toxicity. Only 3 out of 79 evaluable patients experienced local failure as first event. TREC is a phase II UK study evaluating the feasibility of randomising patients to receive either organ-saving treatment with SCRT and TEM versus standard TME surgery. This study is due to report in 2017 having completed minimum 2-year follow-up.

In conclusion, several strategies can be followed to improve the quality of life of patients with rectal cancer by aiming for organ preservation. However, all data so far are derived from small phase II studies and many questions regarding the optimal radiotherapy schedule and the optimal timing of evaluation remain. In addition, prospective comparative data with radical surgery are not available. Therefore, there is an urgent need for a randomised phase III trial to establish the risks, complication rates and benefits of organ saving compared with standard radical surgery for early-stage rectal cancer. The aim of STAR-TREC study is to assess the feasibility of successfully recruiting to a large, multicentre randomised trial comparing radical surgery versus organ saving treatment using (chemo)radiotherapy followed by selective transanal microsurgery.

## Methods and analysis

### Design

STAR-TREC trial is a multicentre international randomised, three-arm parallel study in patients with biopsy-proven adenocarcinoma of the rectum. The trial is coordinated from Birmingham, UK with national hubs in Radboudumc (the Netherlands) and Odense University Hospital Svendborg UMC (Denmark). Participants are currently being recruited and enrolled; the first patient was enrolled in July 2017.

The primary endpoints of STAR-TREC study (phase II) are defined as:

1. Year 1: Randomise at least four cases per month internationally (n=48).
2. Year 2: Randomise at least six cases per month internationally (n=72).

The secondary endpoints of this phase II trial are:

1. Year 1: Can one international partner procure independent funding in year 1? Successful international collaboration will be necessary to deliver a future phase III study.
2. Year 1: Can one international partner open the study to recruit in year 1?
3. Efficacy of organ-preserving treatment arms on completion of phase II study: Is the organ-saving rate >50% at 12 months (following randomisation) in the experimental arms?

Additional outcome measures pertinent to a future phase III study examining the safety and efficacy of organ saving versus standard surgery will also be collected.

#### Safety

- accuracy of MRI in predicting STAR-TREC eligibility;
- 30-day and 6-month mortality;
- surgical morbidity;
- rate of tumour recurrence or regrowth within the bowel wall (experimental arm);
- rate of tumour recurrence within the mesorectum (experimental arm);
- rate of distant metastases;
- pelvic failure rate: expressed as a sum of the following: (1) unresectable pelvic tumour, (2) cases requiring beyond TME surgery or (3) tumour recurrence or regrowth ≤1 mm from the circumferential surgical margin after TME surgery. This outcome measure will be pivotal in challenging current clinical practice and it is our intention that it becomes the primary endpoint in phase III;
- bowel, bladder and sexual dysfunction (baseline and 12, 24 months postrandomisation).

#### Efficacy

- proportion of patients with/without a stoma at 30 days and 1 year;
- histopathological assessment of tumour downstaging following radiotherapy according to depth of tumour invasion and the incidence of other high-risk features in comparison to non-irradiated (control) group;
- proportion of patients identified by clinical and MRI assessment as suitable for active monitoring;
- conversion rates from organ saving to radical surgery;
- disease-free survival;
- quality of life (baseline and 12, 24 months postrandomisation);
- overall survival.

### Study population

This is a hospital-based study. Centre eligibility depends on a Radiotherapy Trials Quality Assurance which is a mixture of an approved departmental standard operating procedure and successful contouring of a case using the new principles of mesorectal irradiation. Candidates will generally be identified in the endoscopy suite following referral for: (1) the investigation of new bowel symptoms, (2) as part of a personal bowel surveillance programme or (3) through national bowel screening. Subjects will then be referred on to either a colorectal surgeon or the colorectal cancer multidisciplinary team (MDT) meeting. Eligibility will be confirmed at the MDT meeting. The main inclusion and exclusion criteria for the trial are summarised in table 1.

**Table 1:** Inclusion and exclusion criteria

Inclusion criteria	Exclusion criteria
1. Age >16 years (UK), age >18 years (Netherlands and Denmark)	1. MRI node positive*
2. Biopsy-proven adenocarcinoma of the rectum	2. MRI extramural invasion present*
3. MRI T1–3b N0 M0 rectal tumour	3. MRI-defined mucinous tumour
4. Multidisciplinary team meeting determines that the following treatment options are all reasonable and feasible: TME surgery, Chemoradiation therapy, Short-course chemoradiation therapy TEM	4. Mesorectal fascia threatened by tumour (≤1 mm on MRI)
5. Estimated creatinine clearance >50 mL/min	5. Maximum tumour diameter >40 mm; measured from everted edges on sagittal MRI
	6. Anterior tumour location above the peritoneal reflection on MRI or endoscopic rectal ultrasound
	7. No residual luminal tumour following endoscopic mucosal resection
	8. Prior pelvic radiotherapy
	9. Regional or distant metastases

*Defined by protocol guidelines.

TEM, transanal endoscopic microsurgery; TME, total mesorectal excision.

### Study arms

**Figure 1:**
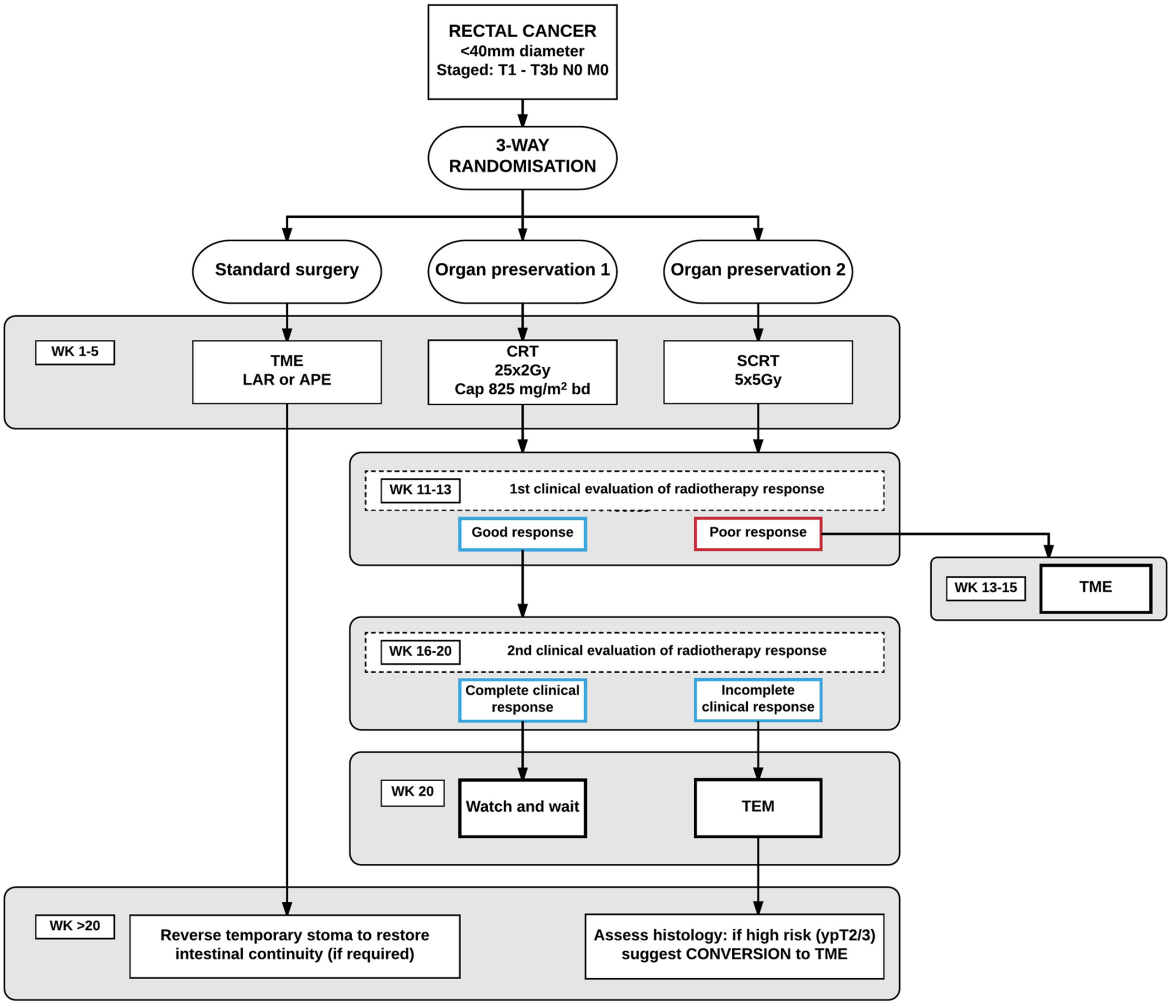
Flow chart of the inclusion, randomisation and management of the study subjects in STAR-TREC trial. APE, anterior perianal excision; CRT, chemoradiation therapy; LAR, low anterior resection; SCRT, short-course radiation therapy; TEM, transanal endoscopic microsurgery; TME, total mesorectal excision.

Patients will be randomised to either standard TME surgery (control), organ-saving treatment using long-course concurrent CRT or organ-saving treatment using SCRT (figure 1). Patients allocated to TME surgery will have a minimum of one abdominal CT scan and regular clinical follow-up will be made according to national guidelines. In the two organ-saving arms, response assessment will take place at 11–13 weeks from the start of (chemo)radiotherapy and again at 16–20 weeks from start. Initial assessment at 11–13 weeks (MRI and endoscopy) will identify a small proportion of cases where radiotherapy has had little or no impact on tumour dimensions. Non-responding patients will be advised to convert to standard TME surgery. Individuals whose tumours demonstrate a satisfactory response at this time point will be examined once again at 16–20 weeks (endoscopy) to determine if a cCR has occurred. It is anticipated that this interval between assessments will allow for additional tumour regression and resolution of acute radiotherapy reactions, facilitating more precise diagnosis of cCR. An active surveillance regime will be performed in the case of a cCR. In the case of incomplete clinical regression, patients will progress to local excision, see figure 1. Representative endoscopic images will be centrally reviewed during this feasibility stage to develop a consistent approach to interpretation of the clinical assessment.

All patients must be assigned to one of the three treatment groups by week 20:

1. Poor response assessed at 11–13 weeks—patient recommended to convert to radical TME surgery.
2. cCR assessed at 16–20 weeks means that the bowel wall has reverted to normal and patients are treated by watchful waiting.
3. Clinically satisfactory, yet incomplete tumour response at 16–20 weeks, meaning a 50% or more reduction of tumour size and the presence of any residual mucosal or bowel wall abnormality suggestive of persisting tumour, will prompt local excision by TEM.

### Treatment regimen for organ-saving strategies

Long-course CRT consists of capecitabine and is administered at a dose of 825 mg/m^2^ two times per day on radiotherapy days only. A total dose of 50 Gy will be applied to the primary tumour and surrounding mesorectum, in 25 fractions of 2 Gy, 5 days per week.

SCRT consists of a total dose of 25 Gy, applied to the primary tumour and surrounding mesorectum in five fractions of 5 Gy, preferably on five consecutive days. Radiotherapy for organ preservation is primarily aimed at tumour downstaging and can therefore be restricted to the peritumoral area including the primary tumour and the mesorectum resulting in a significant reduction in the irradiated target volume.

### Randomisation

Patients will be randomised on a 1:1:1 basis between standard surgical treatment and organ-saving treatments. Randomisation will be provided by a computer-generated program at the University of Birmingham Clinical Trials Units. The randomisation program will use a minimisation procedure for the following variables:

1. MRI tumour staging (≤T3a N0 V0 and T3b N0 V0) (T3a: tumour extends <1 mm beyond muscularis propria; T3b: tumour extends 1–5 mm beyond muscularis propria)
2. country (UK, the Netherlands, Denmark).

Stratification and minimisation will be by T-stage to ensure that the more advanced tumours are equally represented across treatments; stratification and by country will be done to account for any bias arising from any possible differences in pretreatment MRI-based staging assessment.

To avoid any possibility of the treatment allocation becoming too predictable, a random factor will be included within the algorithm whereby for a proportion of the allocations true randomisation will be implemented rather than by using the minimisation allocation.

### Sample size

No power calculation is provided as the primary objective is to show feasibility of recruitment. The aim of the present trial is to include four to six patients per month in order to have a high enough randomisation rate to perform a phase III trial. For a phase III trial, the primary outcome would be 3-year pelvic failure. The null hypothesis is that the increase in the rate of pelvic failure at 3 years with organ preservation compared with standard surgery is less than 7% absolute difference. Prior data indicate that the pelvic recurrence rate in the radical TME group is 2%. If the true recurrence rate for patients in an experimental arm is 9% then, using 90% power and alpha=0.025 (to account for two treatment comparisons) would require 117 patients per treatment arm. Anticipating a 10% dropout rate, we would aim to randomise 400 participants. The final decision for a phase III sample size will be taken from information gained during the feasibility study. Data of the phase II trial will be used for the phase III trial.

### Data management

Case report forms (CRFs) can be entered online at http://www.bctu.bham.ac.uk/STAR-TREC. Authorised staff at sites will require an individual secure login username and password to access this online data entry system. Paper CRFs must be completed, signed/dated and returned to the National STAR-TREC Trial Office by the investigator or an authorised member of the site research team. Data reported on each CRF should be consistent with the source data or the discrepancies should be explained. If information is not known, this must be clearly indicated on the CRF. All missing and ambiguous data will be queried. All sections are to be completed.

Assessment of the health-related quality of life will be done after the patients have completed a series of questionnaires. The questionnaires European Organisation for Research and Treatment of Cancer Quality of life questionnaires for colorectal cancer quality of life questionnaire C30 and CR29 (EORTC QLQL), standardised questionnaire for use as a measure of health outcome (EQ-5D), Low Anterior Resection Syndrome (LARS) score and Patient-completed questionnaire for evaluating male/female lower urinary tract symptoms and impact on quality of life (ICIQ-MLUTS/ICIQ FLUTS) will be done at three time points, at baseline prior to treatment and at follow-up 12 and 24 months after the start of treatment.

All trial records must be archived and securely retained for at least 25 years. No documents will be destroyed without prior approval from the Sponsor, via the central STAR-TREC trial office. On-site monitoring will be carried out as required following a risk assessment and as documented in the monitoring plan for each participating country. Any monitoring activities will be reported to the central STAR-TREC office and any issues noted will be followed up to resolution. STAR-TREC will also be centrally monitored; however, additional on-site monitoring may occur if triggered. Further information regarding data management is provided in the study protocol.

### Ethics and dissemination

The trial will be performed in accordance with the recommendations guiding physicians in biomedical research involving human subjects, adopted by the 18th World Medical Association General Assembly, Helsinki, Finland and stated in the respective participating countries laws governing human research, and Good Clinical Practice. The medical ethical committees of all the participating countries have approved the study protocol.

A meeting will be held after the end of the study to allow discussion of the main results among the collaborators prior to publication. Results of the primary and secondary endpoints will be submitted for publication in peer-reviewed journals.

## Discussion

The TREC and CARTS groups have combined with colleagues in Denmark to design STAR-TREC study. Phase II data from TREC and CARTS justifies a randomised comparison of standard radical surgery versus organ-saving treatment using either SCRT or CRT with selective use of transanal microsurgery based on a radiotherapy response assessment. Organ preservation is not standard treatment and testing the feasibility is important to determine the scale of randomised trial that can be performed. The phase II STAR-TREC study will evaluate the feasibility of accelerating recruitment to an international three-arm randomised trial.

The published literature supports use of (chemo)radiotherapy and transanal microsurgery as an alternative to major surgery for curative treatment of early rectal cancer. To date, studies have recruited patients who were highly motivated to organ-preserving treatment. Broader patient populations are yet to be evaluated using these organ-saving treatments. In addition, the long-term impact of organ-saving treatment after neoadjuvant treatment, on quality of life and, more importantly, oncological outcome is unknown. Therefore, these organ-preserving strategies should ideally be compared with radical TME surgery which represents the current standard of care for patients with rectal cancer. A randomised trial comparing organ-saving treatment with major surgery might be practice changing for the treatment of patients with early rectal cancer.

In addition, while it seems probable that a strategy of organ saving may produce substantial benefits over conventional radical surgery, the optimum organ-saving treatment schedule remains unclear. Phase II studies suggest that SCRT may have the lowest acute toxicity while CRT may achieve the highest cCR rates. Randomisation between these two strategies with the interval calculated from the start of (chemo)radiotherapy will give insight in the possible difference in efficacy in these early cancers. STAR-TREC is, therefore, an international, multicentre, randomised, phase II feasibility study comprising a 1:1:1 randomisation for eligible subjects with early clinically localised rectal cancer.

While published data supports further evaluation of organ saving in patients with early-stage rectal cancer using either SCRT or CRT followed by transanal microsurgery, it has also become clear that not all patients require surgery. Watchful waiting after complete response is being investigated in patients already in need of CRT.^24 26 37^ Other studies introduce (chemo)radiation therapy to the treatment regimen in order to facilitate organ preservation. Current techniques use either SCRT (five fractions of 5 Gy)^40 42^ or concurrent fluoropyridine-based CRT (25 fractions of 1.8 or 2 Gy).^38–40 42^ Radiotherapy is routinely followed by TEM, to remove the portion of bowel wall affected by cancer. However, in a significant proportion of patients, there are no signs of residual tumour following radiotherapy. This is termed a cCR. These patients are likely overtreated by routine transanal microsurgery and therefore possibly subjected to unnecessary surgery-related morbidity. Therefore, patients with a cCR might be better served by a watchful waiting approach.^44^

STAR-TREC study design incorporates several developmental steps, each intended to further reduce treatment-related side effects associated with organ-preserving therapy:

1. Modification in the capecitabine dose from 825 mg/m^2^ two times per day for 7 days per week used in CARTS to 825 mg/m^2^ two times per day for 5 days per week.
2. Use of a smaller radiotherapy volume incorporating only the primary tumour, rectal wall and mesorectum.
3. Use of a two-step clinical response assessment tool following (chemo)radiation so that (1) poor responders are converted to radical TME surgery at the earliest opportunity while (2) good responders are given more time to determine if they reach cCR and may avoid transanal microsurgery.
4. Selective use of transanal microsurgery/TEM for residual mucosal or bowel wall abnormality suggestive of persisting cancer.
5. Objective comparison of the efficacy of CRT versus SCRT with similar intervals between start of radiotherapy and evaluation.

To date, no significant differences are considered for target volume definition for early or advanced rectal cancers. Target volumes contain at least the primary tumour, the mesorectal fat, presacral and internal iliac nodes.^45^ Given that patients in STAR-TREC will be clinically node negative, the necessity of irradiating presacral and iliac nodes is questionable. Even in the case of unexpected nodal involvement, the majority of involved lymph nodes will be peritumoral in the mesorectum, as demonstrated in a series of 121 patients with locally advanced rectal cancer who underwent CRT.^46^ The radiotherapy volume has therefore been reduced to the mesorectal fat only. To ensure safe introduction of this new technique, strict radiotherapy quality assurance is part of the protocol.

STAR-TREC is designed to achieve a recruitment rate that would provide confidence that extension into a phase III trial is achievable. Further applications for funding, ethics approval and a substantial protocol amendment would be required for this transition.

## Conclusion

There is an urgent need for a randomised phase III trial to establish the risks and benefits of organ saving compared with standard TME surgery for early-stage rectal cancer. STAR-TREC trial builds on experience gained through the TREC and CARTS phase II studies. STAR-TREC is a multicentre international randomised phase II study designed to assess the feasibility of recruiting six international patients per month in order to facilitate the evaluation of TME surgery versus organ-saving strategy preceded by (chemo)radiation in two different fractionation schedules. The trial aims to improve the rate of patient recruitment compared with earlier studies and will also introduce a mesorectal target volume with quality assurance. The ultimate goal of this phase II feasibility study is to accelerate to a phase III study comparing TME surgery with two organ-saving treatment regimens.

## Supplementary Material

Reviewer comments

Author's manuscript
