# Supplementary material for: Can we Save the rectum by watchful waiting or TransAnal microsurgery following (chemo) Radiotherapy versus Total mesorectal excision for early REctal Cancer (STAR-TREC study)?: protocol for a multicentre, randomised feasibility study
Source: BMJ Open. 2017 Dec 28;7(12):e019474. doi: 10.1136/bmjopen-2017-019474 (PMC5770914; doi:10.1136/bmjopen-2017-019474)
Supplement: Author's manuscript [file bmjopen-2017-019474.draft_revisions.pdf]

BMJ Open is committed to open peer review. As part of this commitment we make the peer review history of every article we publish publicly available.

When an article is published we post the peer reviewers' comments and the authors' responses online. We also post the versions of the paper that were used during peer review. These are the versions that the peer review comments apply to.

The versions of the paper that follow are the versions that were submitted during the peer review process. They are not the versions of record or the final published versions. They should not be cited or distributed as the published version of this manuscript.

BMJ Open is an open access journal and the full, final, typeset and author-corrected version of record of the manuscript is available on our site with no access controls, subscription charges or pay-per-view fees (http://bmjopen.bmj.com).

If you have any questions on BMJ Open's open peer review process please email editorial.bmjopen@bmj.com

BMJ Open

Can we Save the rectum by watchful waiting or TransAnal microsurgery following (chemo)Radiotherapy versus Total mesorectal excision for early REctal Cancer? – STAR-TREC study: Protocol for a multi-centre, randomised feasibility study

Journal:	*BMJ Open*
Manuscript ID	bmjopen-2017-019474
Article Type:	Protocol
Date Submitted by the Author:	25-Sep-2017
Complete List of Authors:	NA, STAR-TREC Collaborative Group ; Radboudumc
**Primary Subject Heading**:	Surgery
Secondary Subject Heading:	Oncology
Keywords:	rectal cancer, chemoradiation, tem, RADIOTHERAPY, watchful waiting

SCHOLARONE™ Manuscripts

For peer review only

Can we Save the rectum by watchful waiting or TransAnal microsurgery following (chemo)Radiotherapy versus Total mesorectal excision for early REctal Cancer? – STAR-TREC study: Protocol for a multi-centre, randomised feasibility study

Authors: STAR-TREC collaborative group

Collaborators:

Anouk J.M. Rombouts[1], Issam Al-Najami[2], Natalie L. Abbott[3], Ane Appelt[4], Gunnar Baatrup[2], Simon Bach[5], Aneel Bhangu[5], Karen-Lise Garm Spindler[6], Richard Gray[7], Kelly Handley[8], Manjinder Kaur[9], Ellen Kerkhof[10], Camilla Jensenius Kronborg[11], Laura Magill[9], Corrie A.M. Marijnen[10], Iris D Nagtegaal[12], Lars Nyvang[13], Femke P. Peters[10], Per Pfeiffer[14], Cornelis Punt[15], Philip Quirke[16], David Sebag-Montefiore[17], Mark Teo[18], Nick West[16], Johannes HW de Wilt[1]

1. Department of surgery, Radboud University Medical Centre, Nijmegen, Netherlands
2. Department of surgery, Odense University Hospital, Denmark, Aarhus University Hospital, Aarhus, Denmark
3. RTTQA Group, Velindre Cancer Centre, Cardiff, UK
4. Leeds Institute of Cancer and Pathology, University of Leeds, and Leeds Cancer Centre, St. James' University Hospital, UK
5. Department of surgery, University Hospitals Birmingham, Birmingham, UK
6. Department of clinical oncology and department of clinical medicine, Aarhus University Hospital and Aarhus university, Aarhus Denmark
7. Clinical Trial Services Unit, University of Oxford, UK

For peer review only

For peer review only - http://bmjopen.bmj.com/site/about/guidelines.xhtml

8. Institue of Applied Health Research, University of Birmingham Clincial Trials Unit, Birmingham, UK

9. University of Birmingham Clincial Trials Unit, Birmingham, UK

10. Department of radiotherapy, Leiden University Medical Center, Leiden, Netherlands

11. Department of clinical oncology, Aarhus University Hospital, Aarhus, Denmark

12. Department of Pathology, Radboud University Medical Centre, Nijmegen, Netherlands

13. Department of medical physics, Aarhus University Hospital, Aarhus, Denmark

14. Department of oncology, Odense University Hospital, Odense, Denmark

15. Department of medical oncology, Academic Medical Center, Amsterdam, Netherlands

16. Department of pathology, School of Medicine, University of Leeds, UK

17. Department of clinical oncology, Leeds Radiotherapy Research Group, University of Leeds, UK

18. Department of clinical oncology, Leeds Cancer Centre, St James University Hospital, UK

Corresponding author: J.H.W. de Wilt, MD, PhD

Professor of Surgical Oncology

Mailing address: Radboud University Medical Center

Department of Surgery, HP 618

6500 HB Nijmegen

Email: star-trec@radboudumc.nl

Telephone number: 0031 24 361 11 11

For peer review only

For peer review only - http://bmjopen.bmj.com/site/about/guidelines.xhtml

Abstract:

INTRODUCTION: Total mesorectal excision is the highly effective standard treatment for rectal cancer but is associated with significant morbidity and may be over-treatment for low risk cancers. This study is designed to determine the feasibility of international recruitment in a study comparing organ saving approaches versus standard TME surgery.

METHODS AND ANALYSIS: The STAR-TREC trial is a multicentre international randomised, 3 arm-parallel, phase II feasibility study in patients with biopsy proven adenocarcinoma of the rectum. The trial is coordinated from Birmingham UK with national hubs in Radboudumc (The Netherlands) and OUH Svendborg UMC (Denmark). Patients with rectal cancer, staged by CT and MRI as ≤ cT3b (up to 5mm of extramural spread) N0M0 can be included. Patients will be randomised to either standard TME surgery (control), organ saving treatment using long course concurrent chemoradiation, or organ saving treatment using short course radiotherapy. For patients treated with an organ saving strategy, clinical response to (chemo)radiotherapy determines the next treatment step. An active surveillance regime will be performed in the case of a complete clinical regression. In the case of incomplete clinical regression, patients will proceed to local excision using an optimized platform such as transanal endoscopic microsurgery (TEM) or other transanal techniques (e.g. TEO, TAMIS). The primary endpoint of this phase II study is to demonstrate sufficient international recruitment in order to sustain a phase III study incorporating pelvic failure as the primary endpoint. Success in phase II is defined as randomisation of at least 4 cases per month internationally in year 1, rising to at least 6 cases per month internationally during year 2.

ETHICS AND DISSEMINATION: The medical ethical committees of all the participating countries have approved the study protocol. Results of the primary and secondary endpoints

For peer review only - http://bmjopen.bmj.com/site/about/guidelines.xhtml

will be submitted for publication in peer-reviewed journals.

TRIAL REGISTRATION: ISRCTN14240288, ISRCTN registry, 20/10/2016; and NCT02945566, clinicaltrials.gov, oct/2016.

Strengths and limitations of this study

- This phase II study is the first study to randomise between the standard of care in early rectal cancer (i.e. TME surgery) and two organ saving strategies using (chemo) radiotherapy followed by selective transanal microsurgery.

- The STAR TREC study will show whether it is feasible to recruit enough patients for a consecutive international large, multi-centre randomised phase III trial.

- The study design incorporates several adjustments in standard (chemo)radiation therapy protocols intended to reduce treatment related side effects associated with organ saving therapy.

- Clinical nodal staging of rectal cancer is rather unreliable and patients with false negative nodal disease will be included in the study.

- Experience with clinical judgement of a complete response is difficult and needs to be monitored carefully with central reviewing during the study.

For peer review only - http://bmjopen.bmj.com/site/about/guidelines.xhtml

Introduction

The introduction of bowel cancer screening is associated with a significant increase in the incidence of early stage rectal cancer [1, 2]. Total Mesorectal Excision (TME) surgery is an effective oncological treatment for early stage rectal cancer, only 2% and 12% of patients experience local or distant failure respectively [3-5]. However, standard surgery for rectal cancer requires permanent stoma formation in 10-20% of cases and temporary stoma formation in 60-70% [6, 7]. Many temporary stomas are not reversed [8, 9]. Furthermore, TME surgery is associated with major morbidity and mortality in a significant number of patients. Over 50% of all patients following TME surgery experience faecal incontinence, whereas urinary problems and sexual dysfunction are observed in 32-80% of patients [10-14]. Another complication following TME surgery is anastomotic bowel leakage which occurs in approximately 15% of patients [15]. In addition, quality of life studies show that TME surgery is associated with persistently poor social role and body image [12, 16-19]. Mortality following TME surgery rises with age; the six-month mortality following TME surgery is 2.0-4.6 % for young rectal cancer patients and 9.0-13.4% for elderly patients (aged >75 years) [20-22]. There are concerns that TME surgery, which evolved to treat locally advanced, symptomatic tumours, may result in significant over-treatment of early screen-detected tumours. An organ preserving strategy may generate significantly less morbidity without substantially compromising oncological outcomes. Promising outcomes have been reported for (chemo)radiation therapy followed by watchful waiting or local excision.

Habr-Gama's group have notably published a watchful waiting approach to rectal cancer. Of 265 predominantly T3 rectal cancer patients treated with chemoradiotherapy (CRT), 71 patients (27%) had a complete clinical response (cCR) [23, 24]. These patients did not have

For peer review only - http://bmjopen.bmj.com/site/about/guidelines.xhtml

surgery and after a mean follow up of 57 months (range 18-156) only two patients developed local recurrence, one of which was successfully salvaged. A Dutch group then prospectively selected patients with cCR for a watchful waiting strategy (N=21) [25]. After a mean follow-up 25 months (± 19 months), one patient had developed a local recurrence which was salvaged by surgery and all other patients were alive without disease. In 2015, the effect of a radiation boost after CRT was evaluated in a prospective observational Danish study. A watch and wait policy was possible in 40 out of 51 included patients [26]. At one year, local recurrence occurred in 16% of 40 patients who initially showed a cCR. Rectal bleeding was relatively frequent in this study during follow-up perhaps relating to the higher radiotherapy doses that were used. However, these results which combine high cCR rates and low local recurrence rates have not been consistently replicated [27, 28]. Furthermore, CRT is associated with treatment-related morbidity and a mortality-rate of 0.5-1% should be considered [29].

Another organ saving treatment strategy is local excision instead of radical surgery. Early rectal tumours may be locally excised through the anus with low morbidity and mortality using Transanal Endoscopic Microsurgery (TEM), allowing rectal saving treatment [30, 31]. Morbidity and mortality after local excision are much lower than after major resection. Morbidity associated with TEM includes bowel perforation, (transitory) incontinence, wound infection and local pain [32-34]. In a study of 5,305 patients with early-stage rectal cancer, 30-day mortality after local excision was found to be 0.5% compared with 2.4% in patients undergoing major resection (P = 0.008). Morbidity within 30 days of surgery was 4.4% in the local excision group versus 12.7% in the major resection group (P < 0.001) [34]. However, the risk of non-radical resection after local excision is higher and the risks of leaving behind

For peer review only - http://bmjopen.bmj.com/site/about/guidelines.xhtml

For peer review only

microscopic lymph node metastases, is a potential cause of local failure [5, 35]. The incidence of lymph node metastasis ranges from 6% to 14% for T1 tumours, 17% to 23% for T2 tumours, and 49% to 66% for T3 tumours [36].

Combining radiotherapy with TEM surgery could possibly lead to better outcomes because radiotherapy can effectively treat microscopic mesorectal nodal metastases and contribute to tumour downsizing [37, 38]. However, limited prospective evidence currently exists to guide the use of radiotherapy and local excision as curative treatment for early rectal cancer. Lezoche et al randomised 100 patients with T2N0 rectal cancer to chemoradiation therapy (CRT) followed by laparoscopic TME surgery or chemoradiotherapy and TEM with a 6-8 week interval to surgery [39]. After a median follow-up of nine years, local recurrence rates were 6% and 8% in the TME and TEM arms respectively. In a trial of 89 patients with unfavourable cT1N0, cT2N0 or borderline cT2/3N0 tumours by Bujko et al, patients were given neoadjuvant treatment with short course radiation therapy (SCRT) or CRT prior to delayed local excision [40]. No further treatment was offered for good responders, whereas immediate TME surgery was recommended for all other patients. Good responders had a 2-year local recurrence rate of 10%. Of the poor responders, eight patients had a TME and none of this group had a recurrence, however, 18 declined or were unfit for TME, and this group had a 2-year local recurrence rate of 37%. This underlines that in high-risk or poor responding patients, neoadjuvant radiotherapy followed by local excision is inadequate treatment.

CARTS was a non-randomised phase II study that evaluated CRT followed by TEM in 55 patients with stage T1-3N0 rectal cancer [41]. Clinical response was assessed 6-8 weeks after completion of CRT and TEM was performed. Organ saving was achieved in more than half of

For peer review only - http://bmjopen.bmj.com/site/about/guidelines.xhtml

patients and 21 had ypT0 disease. Radiotherapy consisted of 50Gy in 25 fractions and capecitabine 825mg/m2 bid was given for the same period 7 days per week. However, 42% of patients developed at least grade 3 toxicity and there were two toxicity related deaths. A multicenter cohort study from the UK that employed SCRT with TEM after ten weeks demonstrated that 43/62 cases had either no or minimal residual disease following radiotherapy [42]. None of these patients experienced short term pelvic relapse and treatment related toxicity was low. The ACOSOG Z6041 study, a single arm phase II study, evaluated an oxaliplatin and capecitabine concurrent chemotherapy schedule combined with 54Gy of pelvic radiotherapy followed by TEM for T2N0 rectal cancer [43]. Both radiotherapy and chemotherapy schedules required reduction during the study due to acute toxicity. Only 3 out of 79 evaluable patients experienced local failure as first event. TREC is a phase II UK study evaluating the feasibility of randomising patients to receive either organ saving treatment with SCRT and TEM versus standard TME surgery. This study is due to report in 2017 having completed minimum 2 year follow up.

In conclusion, several strategies can be followed to improve the quality of life of rectal cancer patients by aiming for organ preservation. However, all data so far are derived from small phase II studies and many questions regarding the optimal radiotherapy schedule and the optimal timing of evaluation remain. In addition, prospective comparative data with radical surgery are not available. Therefore, there is an urgent need for a randomised phase III trial to establish the risks, complication rates and benefits of organ saving compared to standard radical surgery for early stage rectal cancer. The aim of the STAR-TREC study is to assess the feasibility of successfully recruiting to a large, multi-centre randomised trial

For peer review only - http://bmjopen.bmj.com/site/about/guidelines.xhtml

comparing radical surgery versus organ saving treatment using (chemo) radiotherapy followed by selective transanal microsurgery.

For peer review only

For peer review only - http://bmjopen.bmj.com/site/about/guidelines.xhtml

Methods and analysis

Design

The STAR-TREC trial is a multicentre international randomised, 3 arm-parallel CTIMP study in patients with biopsy proven adenocarcinoma of the rectum. The trial is coordinated from Birmingham UK with national hubs in Radboudumc (The Netherlands) and OUH Svendborg UMC (Denmark). Participants are currently being recruited and enrolled; the first patient was enrolled in July 2017.

The primary endpoints of the STAR TREC study (phase II) are defined as:

1. Year 1: Randomise at least 4 cases per month internationally (n=48);
2. Year 2: Randomise at least 6 cases per month internationally (n=72).

The secondary endpoints of this phase II trial are:

1. Year 1: Can one international partner procure independent funding in year 1? Successful international collaboration will be necessary to deliver a future phase III study.
2. Year 1: Can one international partner open the study to recruit in year 1?
3. Efficacy of organ preserving treatment arms on completion of phase II study: Is the organ saving rate >50% at 12 months (following randomisation) in the experimental arms?

Additional outcome measures pertinent to a future phase III study examining the safety and efficacy of organ saving versus standard surgery will also be collected.

For peer review only - http://bmjopen.bmj.com/site/about/guidelines.xhtml

Safety

- Accuracy of MRI in predicting STAR-TREC eligibility
- 30-day and 6 month mortality
- Surgical morbidity
- Rate of tumour recurrence or regrowth within the bowel wall (experimental arm)
- Rate of tumour recurrence within the mesorectum (experimental arm)
- Rate of distant metastases
- Pelvic failure rate: expressed as a sum of the following (i) unresectable pelvic tumour, (ii) cases requiring beyond TME surgery or (iii) tumour recurrence or regrowth ≤1mm from the circumferential surgical margin after TME surgery. This outcome measure will be pivotal in challenging current clinical practice and it is our intention that it becomes the primary endpoint in phase III.
- Bowel, bladder and sexual dysfunction (baseline and 12, 24 months post randomisation)

Efficacy

- Proportion of patients with/ without a stoma at 30 days and one year

For peer review only - http://bmjopen.bmj.com/site/about/guidelines.xhtml

- Histopathological assessment of tumour down-staging following radiotherapy according to depth of tumour invasion and the incidence of other high-risk features in comparison to non-irradiated (control) group.
- Proportion of patients identified by clinical and MRI assessment as suitable for active monitoring
- Conversion rates from organ saving to radical surgery
- Disease free survival
- Quality of life (baseline and 12, 24 months post randomisation)
- Overall survival

Study population

This is a hospital-based study. Centre eligibility depends on a radiotherapy trials quality assurance (RTTQA) which is a mixture of an approved departmental standard operating procedure and successful contouring of a case using the new principles of mesorectal irradiation. Candidates will generally be identified in the endoscopy suite following referral for (i) the investigation of new bowel symptoms, (ii) as part of a personal bowel surveillance programme or (iii) through national bowel screening. Subjects will then be referred on to either a colorectal surgeon or the colorectal cancer multidisciplinary team (MDT) meeting. Eligibility will be confirmed at the MDT meeting. The main in- and exclusion criteria for the trial are summarized in Table 1.

Study arms

For peer review only - http://bmjopen.bmj.com/site/about/guidelines.xhtml

Patients will be randomised to either standard TME surgery (control), organ saving treatment using long course concurrent CRT or organ saving treatment using SCRT (Figure 1). Patients allocated to TME surgery will have a minimum of one abdominal Computed Tomography (CT) scan and regular clinical follow-up will be made according to national guidelines. In the two organ saving arms, response assessment will take place at 11-13 weeks from the start of (chemo)radiotherapy and again at 16-20 weeks from start. Initial assessment at 11-13 weeks (MRI and endoscopy) will identify a small proportion of cases where radiotherapy has had little or no impact upon tumour dimensions. Non-responding patients will be advised to convert to standard TME surgery. Individuals whose tumours demonstrate a satisfactory response at this time point will be examined once again at 16-20 weeks (endoscopy) to determine if a cCR has occurred. It is anticipated that this interval between assessments will allow for additional tumour regression and resolution of acute radiotherapy reactions, facilitating more precise diagnosis of cCR. An active surveillance regime will be performed in the case of a cCR. In the case of incomplete clinical regression, patients will progress to local excision, see figure 1. Representative endoscopic images will be centrally reviewed during this feasibility stage to develop a consistent approach to interpretation of the clinical assessment.

All patients must be assigned to one of the three treatment groups by week 20:

(1) Poor response assessed at 11-13 weeks – patient recommended to convert to radical TME surgery.

(2) Clinical complete response (cCR) assessed at 16-20 weeks means that the bowel wall has reverted to normal and patients are treated by watchful waiting.

For peer review only - http://bmjopen.bmj.com/site/about/guidelines.xhtml

(3) Clinically satisfactory, yet incomplete tumour response at 16-20 weeks, meaning a 50% or more reduction of tumour size and the presence of any residual mucosal or bowel wall abnormality suggestive of persisting tumour, will prompt local excision by TEM.

Treatment regimen for organ saving strategies

Long course CRT consists of capecitabine and is administered at a dose of 825 mg/m2 bid on radiotherapy days only. A total dose of 50 Gy will be applied to the primary tumour and surrounding mesorectum, in 25 fractions of 2 Gy, 5 days per week.

SCRT consists of a total dose of 25 Gy, applied to the primary tumour and surrounding mesorectum in 5 fractions of 5 Gy, preferably on 5 consecutive days. Radiotherapy for organ preservation is primarily aimed at tumour downstaging and can therefore be restricted to the peritumoural area including the primary tumour and the mesorectum resulting in a significant reduction in the irradiated target volume.

Randomisation

Patients will be randomised on a 1:1:1 basis between standard surgical treatment and organ saving treatments. Randomisation will be provided by a computer-generated program at the University of Birmingham Clinical Trials Units (BCTU). The randomisation programme will use a minimisation procedure for the following variables:

1. MRI Tumour staging (≤T3a N0 V0 and T3b N0 V0)

T3a: tumour extends <1 mm beyond muscularis propria

T3b: tumour extends 1-5 mm beyond muscularis propria

2. Country (UK, the Netherlands, Denmark)

For peer review only

Stratification and minimisation will be by T-stage to ensure that the more advanced tumours are equally represented across treatments; stratification and by country will be done to account for any bias arising from any possible differences in pre-treatment MRI based staging assessment.

To avoid any possibility of the treatment allocation becoming too predictable, a random factor will be included within the algorithm whereby for a proportion of the allocations true randomisation will be implemented rather than by using the minimisation allocation.

Sample size

No power calculation is provided as the primary objective is to show feasibility of recruitment. The aim of the present trial is to include 4-6 patients per month in order to have a high enough randomisation rate to perform a phase III trial. For a phase III trial the primary outcome would be 3-year pelvic failure. The null hypothesis is that the increase in the rate of pelvic failure at 3 years with organ preservation compared to standard surgery is less than 7% absolute difference. Prior data indicate that the pelvic recurrence rate in the radical TME group is 2%. If the true recurrence rate for patients in an experimental arm is 9% then, using 90% power and alpha=0.025 (to account for two treatment comparisons) would require 117 patients per treatment arm. Anticipating a 10% dropout rate, we would aim to randomise 400 participants. The final decision for a phase III sample size will be taken from information gained during the feasibility study. Data of the phase II trial will be used for the phase III trial.

For peer review only - http://bmjopen.bmj.com/site/about/guidelines.xhtml

Data management

Case report forms (CRF) can be entered online at http://www.bctu.bham.ac.uk/STAR-TREC. Authorised staff at sites will require an individual secure login username and password to access this online data entry system. Paper CRFs must be completed, signed/dated and returned to the National STAR-TREC Trial Office by the Investigator or an authorised member of the site research team. Data reported on each CRF should be consistent with the source data or the discrepancies should be explained. If information is not known, this must be clearly indicated on the CRF. All missing and ambiguous data will be queried. All sections are to be completed.

Assessment of the health related quality of life will be done after the patients have completed a series of questionnaires. The questionnaires EORTC QLQ C30 and CR29, EQ-5D, LARS score and ICIQ-MLUTS/ ICIQ-FLUTS will be done at three time points, at baseline prior to treatment and at follow-up 12 and 24 months after the start of treatment.

All trial records must be archived and securely retained for at least 25 years. No documents will be destroyed without prior approval from the Sponsor, via the Central STAR-TREC Trial Office. On-site monitoring will be carried out as required following a risk assessment and as documented in the monitoring plan for each participating country. Any monitoring activities will be reported to the central STAR-TREC office and any issues noted will be followed up to resolution. STAR-TREC will also be centrally monitored; however additional on-site monitoring may occur if triggered. Further information regarding data management is provided in the study protocol.

For peer review only - http://bmjopen.bmj.com/site/about/guidelines.xhtml

Ethics and dissemination

The trial will be performed in accordance with the recommendations guiding physicians in biomedical research involving human subjects, adopted by the 18th World Medical Association General Assembly, Helsinki, Finland, and stated in the respective participating countries laws governing human research, and Good Clinical Practice. The medical ethical committees of all the participating countries have approved the study protocol.

A meeting will be held after the end of the study to allow discussion of the main results among the collaborators prior to publication. Results of the primary and secondary endpoints will be submitted for publication in peer-reviewed journals.

For peer review only

For peer review only - http://bmjopen.bmj.com/site/about/guidelines.xhtml

Discussion

The TREC and CARTS groups have combined with colleagues in Denmark to design the STAR-TREC study. Phase II data from TREC and CARTS justifies a randomised comparison of standard radical surgery versus organ saving treatment using either SCRT or CRT with selective use of transanal microsurgery based upon a radiotherapy response assessment. Organ preservation is not standard treatment and testing the feasibility is important to determine the scale of randomised trial that can be performed. The phase II STAR-TREC study will evaluate the feasibility of accelerating recruitment to an international three arm randomised trial.

The published literature supports use of (chemo)radiotherapy and transanal microsurgery as an alternative to major surgery for curative treatment of early rectal cancer. To date, studies have recruited patients who were highly motivated to organ preserving treatment. Broader patient populations are yet to be evaluated using these organ saving treatments. In addition, the long term impact of organ saving treatment after neoadjuvant treatment, upon quality of life and, more importantly, oncological outcome is unknown. Therefore, these organ preserving strategies should ideally be compared to radical TME surgery which represents the current standard of care for patients with rectal cancer. A randomised trial comparing organ saving treatment with major surgery might be practice changing for the treatment of early rectal cancer patients.

In addition, while it seems probable that a strategy of organ saving may produce substantial benefits over conventional radical surgery, the optimum organ saving treatment schedule remains unclear. Phase II studies suggest that SCRT may have the lowest acute toxicity while

For peer review only - http://bmjopen.bmj.com/site/about/guidelines.xhtml

CRT may achieve the highest cCR rates. Randomisation between these two strategies with the interval calculated from the start of (chemo)radiotherapy will give insight in the possible difference in efficacy in these early cancers. STAR-TREC is therefore an international, multi-centre, randomised, phase II feasibility study comprising a 1:1:1 randomisation for eligible subjects with early clinically localized rectal cancer.

While published data supports further evaluation of organ saving in patients with early stage rectal cancer utilizing either SCRT or CRT followed by transanal microsurgery, it has also become clear that not all patients require surgery. Watchful waiting after complete response is being investigated in patients already in need of CRT [24, 26, 37]. Other studies introduce (chemo)radiation therapy to the treatment regimen in order to facilitate organ preservation. Current techniques utilise either SCRT (5 fractions of 5 Gy) [40, 42] or concurrent fluoropyridine based CRT (25 fractions of 1.8 or 2 Gy) [38-40, 42]. Radiotherapy is routinely followed by TEM, to remove the portion of bowel wall affected by cancer. However, in a significant proportion of patients, there are no signs of residual tumour following radiotherapy. This is termed a cCR. These patients are likely over-treated by routine transanal microsurgery and therefore possibly subjected to unnecessary surgery related morbidity. Therefore, patients with a cCR might be better served by a watchful waiting approach [44].

For peer review only

The STAR-TREC study design incorporates several developmental steps, each intended to further reduce treatment related side effects associated with organ preserving therapy:

1. Modification in the capecitabine dose from 825mg/m2 bd 7 days per week used in CARTS to 825 mg/m2 bid 5 days per week.

For peer review only - http://bmjopen.bmj.com/site/about/guidelines.xhtml

2. Use of a smaller radiotherapy volume incorporating only the primary tumour, rectal wall and mesorectum.

3. Use of a two-step clinical response assessment tool following (chemo)radiation so that (i) poor responders are converted to radical TME surgery at the earliest opportunity while (ii) good responders are given more time to determine if they reach complete clinical response and may avoid transanal microsurgery.

4. Selective use of transanal microsurgery/TEM for residual mucosal or bowel wall abnormality suggestive of persisting cancer.

5. Objective comparison of the efficacy of CRT versus SCRT with similar intervals between start of radiotherapy and evaluation.

To date, no significant differences are considered for target volume definition for early or advanced rectal cancers. Target volumes contain at least the primary tumour, the mesorectal fat, presacral and internal iliac nodes [45]. Given that patients in STAR-TREC will be clinically node negative, the necessity of irradiating presacral and iliac nodes is questionable. Even in the case of unexpected nodal involvement, the majority of involved lymphnodes will be peritumoural in the mesorectum, as demonstrated in a series of 121 patients with locally advanced rectal cancer who underwent chemoradiotherapy [46]. The radiotherapy volume has therefore been reduced to the mesorectal fat only. To ensure safe introduction of this new technique, strict radiotherapy quality assurance is part of the protocol.

STAR-TREC is designed to achieve a recruitment rate that would provide confidence that extension into a phase III trial is achievable. Further applications for funding, ethics approval and a substantial protocol amendment would be required for this transition.

For peer review only - http://bmjopen.bmj.com/site/about/guidelines.xhtml

For peer review only

Conclusion

There is an urgent need for a randomised phase III trial to establish the risks and benefits of organ saving compared to standard TME surgery for early stage rectal cancer. The STAR-TREC trial builds on experience gained through the TREC and CARTS phase II studies. STAR-TREC is a multicenter international randomised phase II study designed to assess the feasibility of recruiting 6 international patients per month in order to facilitate the evaluation of TME surgery versus organ saving strategy preceded by (chemo)radiation in two different fractionation schedules. The trial aims to improve the rate of patient recruitment compared to earlier studies and will also introduce a mesorectal target volume with quality assurance. The ultimate goal of this phase II feasibility study is to accelerate to a phase III study comparing TME surgery with two organ saving treatment regimens.

For peer review only - http://bmjopen.bmj.com/site/about/guidelines.xhtml

References

1. Atkin WS, Cook CF, Cuzick J, Edwards R, Northover JM, Wardle J. Single flexible sigmoidoscopy screening to prevent colorectal cancer: baseline findings of a UK multicentre randomised trial. Lancet. 2002;359(9314):1291-1300.

2. Results of the first round of a demonstration pilot of screening for colorectal cancer in the United Kingdom. BMJ. 2004;329(7458):133.

3. Bentrem DJ, Okabe S, Wong WD, Guillem JG, Weiser MR, Temple LK, et al. T1 adenocarcinoma of the rectum: transanal excision or radical surgery? Ann Surg. 2005;242(4):472-479.

4. Peeters KC, Marijnen CA, Nagtegaal ID, Kranenbarg EK, Putter H, Wiggers T, Rutten H, Pahlman L, Glimelius B, Leer JW et al: The TME trial after a median follow-up of 6 years: increased local control but no survival benefit in irradiated patients with resectable rectal carcinoma. Ann Surg. 2007;246(5):693-701.

5. Endreseth BH, Myrvold HE, Romundstad P, Hestvik UE, Bjerkeset T, Wibe A, et al. Transanal excision vs. major surgery for T1 rectal cancer. Dis Colon Rectum. 2005;48(7):1380-1388.

6. Bokkerink GM, de Graaf EJ, Punt CJ, Nagtegaal ID, Rutten H, Nuyttens JJ, et al. The CARTS study: Chemoradiation therapy for rectal cancer in the distal rectum followed by organ-sparing transanal endoscopic microsurgery. BMC Surg. 2011;11:34.

7. Homan J, Bokkerink GM, Aarts MJ, Lemmens VE, van Lijnschoten G, Rutten HJ, et al. Variation in circumferential resection margin: Reporting and involvement in the South-Netherlands. Eur J Surg Oncol. 2015;41(11):1485-1492.

For peer review only

For peer review only - http://bmjopen.bmj.com/site/about/guidelines.xhtml

8. Anderin K, Gustafsson UO, Thorell A, Nygren J. The effect of diverting stoma on long-term morbidity and risk for permanent stoma after low anterior resection for rectal cancer. Eur J Surg Oncol. 2016;42(6):788-793.

9. Kim MJ, Kim YS, Park SC, Sohn DK, Kim DY, Chang HJ, et al. Risk factors for permanent stoma after rectal cancer surgery with temporary ileostomy. Surgery. 2016;159(3):721-727.

10. Hendren SK, O'Connor BI, Liu M, Asano T, Cohen Z, Swallow CJ, et al. Prevalence of male and female sexual dysfunction is high following surgery for rectal cancer. Ann Surg. 2005;242(2):212-223.

11. Wallner C, Lange MM, Bonsing BA, Maas CP, Wallace CN, Dabhoiwala NF, et al. Causes of fecal and urinary incontinence after total mesorectal excision for rectal cancer based on cadaveric surgery: a study from the Cooperative Clinical Investigators of the Dutch total mesorectal excision trial. J Clin Oncol. 2008;26(27):4466-4472.

12. Engel J, Kerr J, Schlesinger-Raab A, Eckel R, Sauer H, Holzel D. Quality of life in rectal cancer patients: a four-year prospective study. Ann Surg. 2003;238(2):203-213.

13. Temple LK, Bacik J, Savatta SG, Gottesman L, Paty PB, Weiser MR, et al. The development of a validated instrument to evaluate bowel function after sphincter-preserving surgery for rectal cancer. Dis Colon Rectum. 2005;48(7):1353-1365.

14. Lange MM, Marijnen CA, Maas CP, Putter H, Rutten HJ, Stiggelbout AM, et al. Risk factors for sexual dysfunction after rectal cancer treatment. Eur J Cancer. 2009;45(9):1578-88.

For peer review only - http://bmjopen.bmj.com/site/about/guidelines.xhtml

15. Marijnen CA, Kapiteijn E, van de Velde CJ, Martijn H, Steup WH, Wiggers T, et al. Acute side effects and complications after short-term preoperative radiotherapy combined with total mesorectal excision in primary rectal cancer: report of a multicenter randomized trial. J Clin Oncol. 2002;20(3):817-825.

16. Grumann MM, Noack EM, Hoffmann IA, Schlag PM. Comparison of quality of life in patients undergoing abdominoperineal extirpation or anterior resection for rectal cancer. Ann Surg. 2001;233(2):149-156.

17. Wilson TR, Alexander DJ. Clinical and non-clinical factors influencing postoperative health-related quality of life in patients with colorectal cancer. Br J Surg. 2008;95(11):1408-1415.

18. Stephens RJ, Thompson LC, Quirke P, Steele R, Grieve R, Couture J, et al. Impact of short-course preoperative radiotherapy for rectal cancer on patients' quality of life: data from the Medical Research Council CR07/National Cancer Institute of Canada Clinical Trials Group C016 randomized clinical trial. J Clin Oncol. 2010;28(27):4233-4239.

19. Wiltink LM, Marijnen CA, Meershoek-Klein Kranenbarg E, van de Velde CJ, Nout RA. A comprehensive longitudinal overview of health-related quality of life and symptoms after treatment for rectal cancer in the TME trial. Acta Oncol. 2016;55(4):502-508.

20. Kapiteijn E, Marijnen CA, Nagtegaal ID, Putter H, Steup WH, Wiggers T, et al. Preoperative radiotherapy combined with total mesorectal excision for resectable rectal cancer. N Engl J Med. 2001;345(9):638-646.

21. Tekkis PP, Poloniecki JD, Thompson MR, Stamatakis JD. Operative mortality in colorectal cancer: prospective national study. BMJ. 2003;327(7425):1196-1201.

For peer review only - http://bmjopen.bmj.com/site/about/guidelines.xhtml

22. Bos AC, van Erning FN, Elferink MA, Rutten HJ, van Oijen MG, de Wilt JH, et al. No Difference in Overall Survival Between Hospital Volumes for Patients With Colorectal Cancer in The Netherlands. Dis Colon Rectum. 2016;59(10):943-952.

23. Habr-Gama A, Gama-Rodrigues J, Sao Juliao GP, Proscurshim I, Sabbagh C, Lynn PB, et al. Local recurrence after complete clinical response and watch and wait in rectal cancer after neoadjuvant chemoradiation: impact of salvage therapy on local disease control. Int J Radiat Oncol Biol Phys. 2014;88(4):822-828.

24. Habr-Gama A, Perez RO, Nadalin W, Sabbaga J, Ribeiro U, Jr., Silva e Sousa AH, Jr., et al. Operative versus nonoperative treatment for stage 0 distal rectal cancer following chemoradiation therapy: long-term results. Ann Surg. 2004;240(4):711-717.

25. Maas M, Beets-Tan RG, Lambregts DM, Lammering G, Nelemans PJ, Engelen SM, et al. Wait-and-see policy for clinical complete responders after chemoradiation for rectal cancer. J Clin Oncol. 2011;29(35):4633-4640.

26. Appelt AL, Ploen J, Harling H, Jensen FS, Jensen LH, Jorgensen JC, et al. High-dose chemoradiotherapy and watchful waiting for distal rectal cancer: a prospective observational study. Lancet Oncol. 2015;16(8):919-927.

27. Glynne-Jones R, Wallace M, Livingstone JI, Meyrick-Thomas J. Complete clinical response after preoperative chemoradiation in rectal cancer: is a "wait and see" policy justified? Dis Colon Rectum. 2008;51(1):10-19.

28. Renehan AG, Malcomson L, Emsley R, Gollins S, Maw A, Myint AS, et al. Watch-and-wait approach versus surgical resection after chemoradiotherapy for patients with rectal cancer (the OnCoRe project): a propensity-score matched cohort analysis. Lancet Oncol. 2016;17(2):174-183.

For peer review only

For peer review only - http://bmjopen.bmj.com/site/about/guidelines.xhtml

29. Garcia-Aguilar J, Chow OS, Smith DD, Marcet JE, Cataldo PA, Varma MG, et al. Effect of adding mFOLFOX6 after neoadjuvant chemoradiation in locally advanced rectal cancer: a multicentre, phase 2 trial. Lancet Oncol. 2015;16(8):957-966.

30. Bach SP, Hill J, Monson JR, Simson JN, Lane L, Merrie A, Warren B, et al. A predictive model for local recurrence after transanal endoscopic microsurgery for rectal cancer. Br J Surg. 2009;96(3):280-290.

31. Cataldo PA, O'Brien S, Osler T. Transanal endoscopic microsurgery: a prospective evaluation of functional results. Dis Colon Rectum. 2005;48(7):1366-1371.

32. Restivo A, Zorcolo L, D'Alia G, Cocco F, Cossu A, Scintu F, et al. Risk of complications and long-term functional alterations after local excision of rectal tumors with transanal endoscopic microsurgery (TEM). Int J Colorectal Dis. 2016;31(2):257-266.

33. Kumar AS, Coralic J, Kelleher DC, Sidani S, Kolli K, Smith LE. Complications of transanal endoscopic microsurgery are rare and minor: a single institution's analysis and comparison to existing data. Dis Colon Rectum. 2013;56(3):295-300.

34. You YN, Baxter NN, Stewart A, Nelson H. Is the increasing rate of local excision for stage I rectal cancer in the United States justified?: a nationwide cohort study from the National Cancer Database. Ann Surg. 2007;245(5):726-733.

35. Garcia-Aguilar J, Mellgren A, Sirivongs P, Buie D, Madoff RD, Rothenberger DA. Local excision of rectal cancer without adjuvant therapy: a word of caution. Ann Surg. 2000;231(3):345-351.

36. Ricciardi R, Madoff RD, Rothenberger DA, Baxter NN. Population-based analyses of lymph node metastases in colorectal cancer. Clin Gastroenterol Hepatol. 2006;4(12):1522-1527.

For peer review only

37. Martens MH, Maas M, Heijnen LA, Lambregts DM, Leijtens JW, Stassen LP, et al. Long-term Outcome of an Organ Preservation Program After Neoadjuvant Treatment for Rectal Cancer. J Nat Cancer Inst. 2016;108(12).

38. van Gijn W, Marijnen CA, Nagtegaal ID, Kranenbarg EM, Putter H, Wiggers T, et al. Preoperative radiotherapy combined with total mesorectal excision for resectable rectal cancer: 12-year follow-up of the multicentre, randomised controlled TME trial. Lancet Oncol. 2011;12(6):575-582.

39. Lezoche E, Baldarelli M, Lezoche G, Paganini AM, Gesuita R, Guerrieri M. Randomized clinical trial of endoluminal locoregional resection versus laparoscopic total mesorectal excision for T2 rectal cancer after neoadjuvant therapy. Br J Surg. 2012;99(9):1211-1218.

40. Bujko K, Richter P, Smith FM, Polkowski W, Szczepkowski M, Rutkowski A, et al. Preoperative radiotherapy and local excision of rectal cancer with immediate radical re-operation for poor responders: a prospective multicentre study. Radiother Oncol. 2013;106(2):198-205.

41. Verseveld M, de Graaf EJ, Verhoef C, van Meerten E, Punt CJ, de Hingh IH, et al. Chemoradiation therapy for rectal cancer in the distal rectum followed by organ-sparing transanal endoscopic microsurgery (CARTS study). Br J Surg. 2015;102(7):853-860.

42. Smart CJ, Korsgen S, Hill J, Speake D, Levy B, Steward M, et al. Multicentre study of short-course radiotherapy and transanal endoscopic microsurgery for early rectal cancer. Br J Surg. 2016;103(8):1069-1075.

For peer review only - http://bmjopen.bmj.com/site/about/guidelines.xhtml

43. Garcia-Aguilar J, Renfro LA, Chow OS, Shi Q, Carrero XW, Lynn PB, et al. Organ preservation for clinical T2N0 distal rectal cancer using neoadjuvant chemoradiotherapy and local excision (ACOSOG Z6041): results of an open-label, single-arm, multi-institutional, phase 2 trial. Lancet Oncol. 2015;16(15):1537-1546.

44. Marijnen CA. Organ preservation in rectal cancer: have all questions been answered? Lancet Oncol. 2015;16(1):e13-22.

45. Valentini V, Gambacorta MA, Barbaro B, Chiloiro G, Coco C, Das P, et al. International consensus guidelines on Clinical Target Volume delineation in rectal cancer. Radiother Oncol. 2016;120(2):195-201.

46. Leibold T, Shia J, Ruo L, Minsky BD, Akhurst T, Gollub MJ, et al. Prognostic implications of the distribution of lymph node metastases in rectal cancer after neoadjuvant chemoradiotherapy. J Clin Oncol. 2008;26(13):2106-2111.

For peer review only

For peer review only - http://bmjopen.bmj.com/site/about/guidelines.xhtml

List of abbreviations

BCTU - Birmingham Clinical Trials Units; **bid –** twice daily; **CARTS –** Trial: Chemoradiation therapy for rectal cancer in the distal rectum followed by organ-sparing transanal endoscopic microsurgery; **cCR -** complete clinical response; **CRT -** Chemo Radiation Therapy; **CT -** Computed Tomography; **CTIMP -** Clinical Trial of an Investigational Medicinal Product; **EORTC QLQ CR 29&C30 –** European Organisation for Research and Treatment of Cancer Quality of life questionnaires for colorectal cancer; **ERUS –** EndoRectal UltraSound; **ESMO –** European Society for Medical Oncology; **EQ-5D –** standardised questionnaire for use as a measure of health outcome; **Gy -** Grey; **HRQL –** Health Related Quality of Life; **ICIQ-MLUTS -** Patient-completed questionnaire for evaluating male lower urinary tract symptoms and impact on quality of life; **ICIQ-FLUTS -** Patient-completed questionnaire for evaluating female lower urinary tract symptoms and impact on quality of life; **LARS score –** Low Anterior Resection Syndrome score; **MDT -** Multi Disciplinary Team meeting; **MRI -** Magnetic Resonance Image; **MRIemvi -** MRI extramural vascular invasion; **MRItrg -** MRI based tumour regression grade; **RTTQA -** RadioTherapy Trials Quality Assurance; **SCRT -** Short Course Radiation Therapy; **STAR-TREC -** Trial name – Can we Save the rectum by watchful waiting or TransAnal microsurgery following (chemo)Radiotherapy versus Total mesorectal excision for early Rectal Cancer; **TAMIS –** TransAnal Minimally Invasive Surgery; **TEM -** Trans anal Endoscopic Microsurgery; **TEO –** Transanal Endoscopic Operation; **TME -** Total Mesorectal Excision; **TMG –** Trial Management Group; **TREC –** Trial: Transanal endoscopic microsurgery and radiotherapy in early rectal cancer; **UK –** United Kingdom.

For peer review only - http://bmjopen.bmj.com/site/about/guidelines.xhtml

Authors' contributions

All collaborators made substantial contributions to the design of the study and/or were involved in drafting the manuscript. All collaborators read and approved the final manuscript.

Funding

STAR-TREC is an international study, separately funded in each participating country. In the UK, this work was supported by Cancer Research UK (C41557/A19393), in the Netherlands by the Dutch Cancer Society (KWF KUN 2014-7448) and in Denmark by the Danish Cancer Society (R100-A6747).

Competing interests statement

There are no competing interests to declare.

Contact information Trial sponsor

Birmingham Clinical Trials Unit (BCTU)
Institute of Applied Health Research
College of Medical and Dental Sciences
Public Health Building
University of Birmingham
Edgbaston
Birmingham
B15 2TT
United Kingdom
0121 415 9104
STAR-TREC@trials.bham.ac.uk

For peer review only

For peer review only - http://bmjopen.bmj.com/site/about/guidelines.xhtml

Table 1 – Inclusion and exclusion criteria

Inclusion criteria	Exclusion criteria
1. Age >16 years (UK), Age >18 years (Netherlands and Denmark)	1. MRI node positive*
2. Biopsy proven adenocarcinoma of the rectum	2. MRI extramural invasion (mriEMVI) present*
3. Magnetic Resonance Image T1-3b N0 M0 rectal tumour	3. MRI defined mucinous tumour
4. Multi disciplinary team meeting determines that the following treatment options are all reasonable and feasible:<br>(a) TME surgery,<br>(b) Chemo Radiation Therapy,<br>(c) Short Course Chemo Radiation Therapy<br>(d) TEM	4. Mesorectal fascia threatened by tumour (≤ 1mm on MRI)
5. Estimated creatinine clearance >50 ml/min	5. Maximum tumour diameter > 40mm; measured from everted edges on sagittal MRI
	6. Anterior tumour location above the peritoneal reflection on MRI or ERUS
	7. No residual luminal tumour following endoscopic mucosal resection
	8. Prior pelvic radiotherapy
	9. Regional or distant metastases

** Defined by protocol guidelines*

For peer review only - http://bmjopen.bmj.com/site/about/guidelines.xhtml

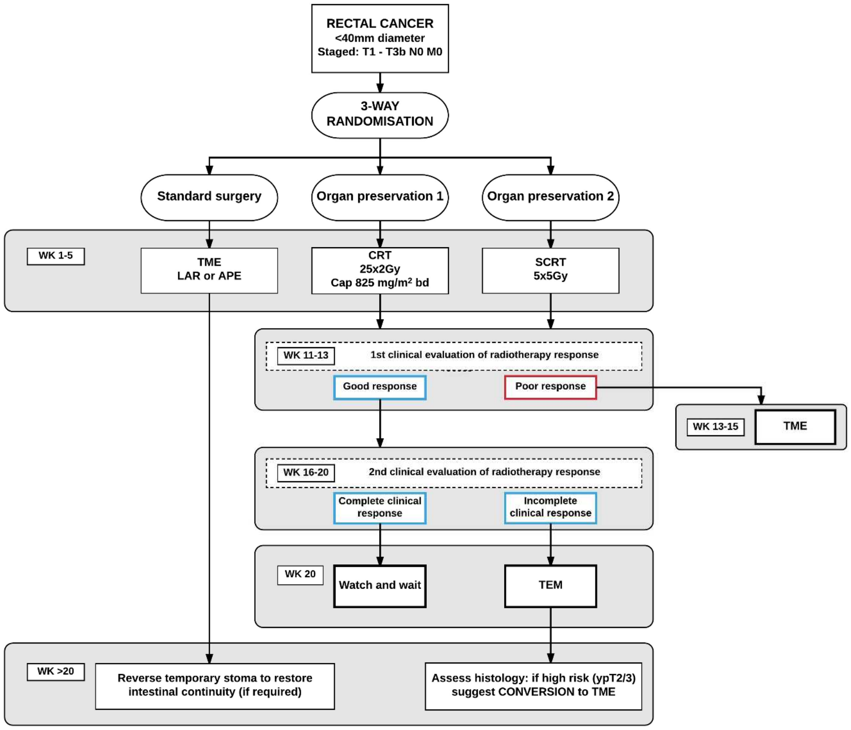

Fig. 1 Flowchart of the inclusion, randomisation and management of the study subjects in the STAR TREC trial. LAR, low anterior resection; APE, anterior perianal excision; TME, total mesorectal excision; CRT, chemoradiation therapy; SCRT, short course primary radiation therapy; TEM, transanal microscopic microsurgery.

279x215mm (300 x 300 DPI)

For peer review only - http://bmjopen.bmj.com/site/about/guidelines.xhtml

SPIRIT

STANDARD PROTOCOL ITEMS: RECOMMENDATIONS FOR INTERVENTIONAL TRIALS

SPIRIT 2013 Checklist: Recommended items to address in a clinical trial protocol and related documents*

Section/item	Item No	Description	Addressed on page number
**Administrative information**			
Title	1	Descriptive title identifying the study design, population, interventions, and, if applicable, trial acronym	Manuscript p1
Trial registration	2a	Trial identifier and registry name. If not yet registered, name of intended registry	Manuscript p 4
	2b	All items from the World Health Organization Trial Registration Data Set	Manuscript p 4, 10-15, 31
Protocol version	3	Date and version identifier	Attachment, p2
Funding	4	Sources and types of financial, material, and other support	Manuscript, p 30
Roles and responsibilities	5a	Names, affiliations, and roles of protocol contributors	Manuscript p 1, 2, 31
	5b	Name and contact information for the trial sponsor	Manuscript p 30
	5c	Role of study sponsor and funders, if any, in study design; collection, management, analysis, and interpretation of data; writing of the report; and the decision to submit the report for publication, including whether they will have ultimate authority over any of these activities	Attachment, p 87-88

For peer review only - http://bmjopen.bmj.com/site/about/guidelines.xhtml

For peer review only

	5d	Composition, roles, and responsibilities of the coordinating centre, steering committee, endpoint adjudication committee, data management team, and other individuals or groups overseeing the trial, if applicable (see Item 21a for data monitoring committee)	Attachment, p 87-88
**Introduction**			
Background and rationale	6a	Description of research question and justification for undertaking the trial, including summary of relevant studies (published and unpublished) examining benefits and harms for each intervention	Manuscript p 5-9
	6b	Explanation for choice of comparators	Manuscript p 5-9
Objectives	7	Specific objectives or hypotheses	Manuscript p 8-9
Trial design	8	Description of trial design including type of trial (eg, parallel group, crossover, factorial, single group), allocation ratio, and framework (eg, superiority, equivalence, noninferiority, exploratory)	Manuscript p 10
**Methods: Participants, interventions, and outcomes**			
Study setting	9	Description of study settings (eg, community clinic, academic hospital) and list of countries where data will be collected. Reference to where list of study sites can be obtained	Manuscript p 10
Eligibility criteria	10	Inclusion and exclusion criteria for participants. If applicable, eligibility criteria for study centres and individuals who will perform the interventions (eg, surgeons, psychotherapists)	Manuscript p 31
Interventions	11a	Interventions for each group with sufficient detail to allow replication, including how and when they will be administered	Manuscript p 13-14
	11b	Criteria for discontinuing or modifying allocated interventions for a given trial participant (eg, drug dose change in response to harms, participant request, or improving/worsening disease)	Manuscript p 13-14
	11c	Strategies to improve adherence to intervention protocols, and any procedures for monitoring adherence (eg, drug tablet return, laboratory tests)	Manuscript p 19-20

For peer review only - http://bmjopen.bmj.com/site/about/guidelines.xhtml

	11d	Relevant concomitant care and interventions that are permitted or prohibited during the trial	Attachment, p53
Outcomes	12	Primary, secondary, and other outcomes, including the specific measurement variable (eg, systolic blood pressure), analysis metric (eg, change from baseline, final value, time to event), method of aggregation (eg, median, proportion), and time point for each outcome. Explanation of the clinical relevance of chosen efficacy and harm outcomes is strongly recommended	Manuscript p 10-12
Participant timeline	13	Time schedule of enrolment, interventions (including any run-ins and washouts), assessments, and visits for participants. A schematic diagram is highly recommended (see Figure)	Figure 1, Manuscript p 12-15
Sample size	14	Estimated number of participants needed to achieve study objectives and how it was determined, including clinical and statistical assumptions supporting any sample size calculations	Manuscript p 15
Recruitment	15	Strategies for achieving adequate participant enrolment to reach target sample size	Manuscript p 12
**Methods: Assignment of interventions (for controlled trials)**			
Allocation:			
Sequence generation	16a	Method of generating the allocation sequence (eg, computer-generated random numbers), and list of any factors for stratification. To reduce predictability of a random sequence, details of any planned restriction (eg, blocking) should be provided in a separate document that is unavailable to those who enrol participants or assign interventions	Manuscript p 14-15
Allocation concealment mechanism	16b	Mechanism of implementing the allocation sequence (eg, central telephone; sequentially numbered, opaque, sealed envelopes), describing any steps to conceal the sequence until interventions are assigned	Manuscript p 14-15, attachment, p43
Implementation	16c	Who will generate the allocation sequence, who will enrol participants, and who will assign participants to interventions	Attachment, p42-43
Blinding (masking)	17a	Who will be blinded after assignment to interventions (eg, trial participants, care providers, outcome assessors, data analysts), and how	Not applicable

For peer review only - http://bmjopen.bmj.com/site/about/guidelines.xhtml

	17b	If blinded, circumstances under which unblinding is permissible, and procedure for revealing a participant's allocated intervention during the trial	Not applicable
**Methods: Data collection, management, and analysis**			
Data collection methods	18a	Plans for assessment and collection of outcome, baseline, and other trial data, including any related processes to promote data quality (eg, duplicate measurements, training of assessors) and a description of study instruments (eg, questionnaires, laboratory tests) along with their reliability and validity, if known. Reference to where data collection forms can be found, if not in the protocol	Manuscript p 16
	18b	Plans to promote participant retention and complete follow-up, including list of any outcome data to be collected for participants who discontinue or deviate from intervention protocols	Attachment, p 70-71
Data management	19	Plans for data entry, coding, security, and storage, including any related processes to promote data quality (eg, double data entry; range checks for data values). Reference to where details of data management procedures can be found, if not in the protocol	Manuscript p 16
Statistical methods	20a	Statistical methods for analysing primary and secondary outcomes. Reference to where other details of the statistical analysis plan can be found, if not in the protocol	Attachment, p 84-87
	20b	Methods for any additional analyses (eg, subgroup and adjusted analyses)	attachment, p 86
	20c	Definition of analysis population relating to protocol non-adherence (eg, as randomised analysis), and any statistical methods to handle missing data (eg, multiple imputation)	attachment, p 84-87
**Methods: Monitoring**			
Data monitoring	21a	Composition of data monitoring committee (DMC); summary of its role and reporting structure; statement of whether it is independent from the sponsor and competing interests; and reference to where further details about its charter can be found, if not in the protocol. Alternatively, an explanation of why a DMC is not needed	Manuscript p 16, attachment p 88
	21b	Description of any interim analyses and stopping guidelines, including who will have access to these interim results and make the final decision to terminate the trial	Attachment, p 86,88

For peer review only - http://bmjopen.bmj.com/site/about/guidelines.xhtml

Harms	22	Plans for collecting, assessing, reporting, and managing solicited and spontaneously reported adverse events and other unintended effects of trial interventions or trial conduct	Attachment, p72-78
Auditing	23	Frequency and procedures for auditing trial conduct, if any, and whether the process will be independent from investigators and the sponsor	Attachment, p82
**Ethics and dissemination**			
Research ethics approval	24	Plans for seeking research ethics committee/institutional review board (REC/IRB) approval	Manuscript p 17
Protocol amendments	25	Plans for communicating important protocol modifications (eg, changes to eligibility criteria, outcomes, analyses) to relevant parties (eg, investigators, REC/IRBs, trial participants, trial registries, journals, regulators)	Attachment, p91
Consent or assent	26a	Who will obtain informed consent or assent from potential trial participants or authorised surrogates, and how (see Item 32)	Attachment, p40-41
	26b	Additional consent provisions for collection and use of participant data and biological specimens in ancillary studies, if applicable	Not applicable
Confidentiality	27	How personal information about potential and enrolled participants will be collected, shared, and maintained in order to protect confidentiality before, during, and after the trial	Manuscript p16
Declaration of interests	28	Financial and other competing interests for principal investigators for the overall trial and each study site	Manuscript p 30
Access to data	29	Statement of who will have access to the final trial dataset, and disclosure of contractual agreements that limit such access for investigators	Attachment, p 78-80 and 89
Ancillary and post-trial care	30	Provisions, if any, for ancillary and post-trial care, and for compensation to those who suffer harm from trial participation	Attachment p 90
Dissemination policy	31a	Plans for investigators and sponsor to communicate trial results to participants, healthcare professionals, the public, and other relevant groups (eg, via publication, reporting in results databases, or other data sharing arrangements), including any publication restrictions	Manuscript p 17

For peer review only - http://bmjopen.bmj.com/site/about/guidelines.xhtml

	31b	Authorship eligibility guidelines and any intended use of professional writers	Attachment p 91
	31c	Plans, if any, for granting public access to the full protocol, participant-level dataset, and statistical code	Full protocol is provided, others not applicable
**Appendices**			
Informed consent materials	32	Model consent form and other related documentation given to participants and authorised surrogates	Attached
Biological specimens	33	Plans for collection, laboratory evaluation, and storage of biological specimens for genetic or molecular analysis in the current trial and for future use in ancillary studies, if applicable	Not applicable

*It is strongly recommended that this checklist be read in conjunction with the SPIRIT 2013 Explanation & Elaboration for important clarification on the items. Amendments to the protocol should be tracked and dated. The SPIRIT checklist is copyrighted by the SPIRIT Group under the Creative Commons "Attribution-NonCommercial-NoDerivs 3.0 Unported" license.

For peer review only
