# Supplementary material for: Can we Save the rectum by watchful waiting or TransAnal microsurgery following (chemo) Radiotherapy versus Total mesorectal excision for early REctal Cancer (STAR-TREC study)?: protocol for a multicentre, randomised feasibility study
Source: BMJ Open. 2017 Dec 28;7(12):e019474. doi: 10.1136/bmjopen-2017-019474 (PMC5770914; doi:10.1136/bmjopen-2017-019474)
Supplement: Reviewer comments [file bmjopen-2017-019474.reviewer_comments.pdf]

PEER REVIEW HISTORY

BMJ Open publishes all reviews undertaken for accepted manuscripts. Reviewers are asked to complete a checklist review form (**http://bmjopen.bmj.com/site/about/resources/checklist.pdf**) and are provided with free text boxes to elaborate on their assessment. These free text comments are reproduced below.

ARTICLE DETAILS

**TITLE (PROVISIONAL)**	Can we Save the rectum by watchful waiting or TransAnal microsurgery following (chemo)Radiotherapy versus Total mesorectal excision for early REctal Cancer? – STAR-TREC study: Protocol for a multi-centre, randomised feasibility study
**AUTHORS**	NA, STAR-TREC Collaborative Group

This article was not externally reviewed at BMJ Open. Protocols that have been independently assessed prior to submission to BMJ Open are usually fast-tracked to publication on the grounds that further substantial changes will not be possible. This independent assessment will usually be external, independent review for both a substantial grant award from a non-commercial or government funder and ethics approval.
